# Neuroimmunomodulation of tissue injury and disease: an expanding view of the inflammatory reflex pathway

**DOI:** 10.1186/s42234-019-0029-8

**Published:** 2019-08-13

**Authors:** Shinji Tanaka, Benjamin Hammond, Diane L. Rosin, Mark D. Okusa

**Affiliations:** 1 Division of Nephrology and Center for Immunity, Inflammation and Regenerative Medicine, University of Virginia, Charlottesville, Virginia USA; 2 Byram Hills High School, Armonk, New York, USA; 3 Department of Pharmacology, University of Virginia, Charlottesville, Virginia USA

**Keywords:** Acute kidney injury, Gut microbiota, Cholinergic anti-inflammatory pathway, Rheumatoid arthritis, Colitis

## Abstract

Neuroimmunomodulation through peripheral nerve activation is an important therapeutic approach to various disorders. Central to this approach is the inflammatory reflex pathway in which the cholinergic anti-inflammatory pathway represents the efferent limb. Recent studies provide a framework for understanding this control pathway, however our understanding remains incomplete. Genetically modified mice, using optogenetics and pharmacogenomics, have been invaluable resources that will allow investigators to disentangle neural pathways that provide a unifying mechanism by which vagal nerve stimulation (and other means of stimulating the pathway) leads to an anti-inflammatory and tissue protective effect. In this review we describe disease models that contribute to our understanding of how vagal nerve stimulation attenuates inflammation and organ injury: acute kidney injury, rheumatoid arthritis, and inflammatory gastrointestinal disease. The gut microbiota contributes to health and disease and the potential role of the vagus nerve in affecting the relationship between gut microbiota and the immune system and modifying diseases remains an intriguing opportunity to attenuate local and systemic inflammation that undergird disease processes.

## Background

A burgeoning field of bioelectronics now offers tools and novel non-pharmacological approaches to activate neuroimmunomodulatory mechanisms for organ protection in diseases and disorders, including hypertension (renal denervation), heart failure, obesity, epilepsy, inflammation, diabetes, bronchoconstriction (forming the basis of anticholinergic treatment of chronic obstructive pulmonary disease), migraines and others. Recently, we reported a simple ultrasound (US)-based protocol that reduced tissue and systemic inflammation and prevented ischemia-reperfusion injury (IRI) in mice (Gigliotti et al. 2013). This effect was dependent on the spleen and functional α7 nicotinic acetylcholine receptors (α7nAChRs), consistent with the hypothesis that US activated the splenic cholinergic anti-inflammatory pathway (CAP) (Tracey 2007). This inflammatory reflex, a neuro-immune circuit, is critical for immunological homeostasis (for recent reviews see (Okusa et al. 2017; Pavlov et al. 2018)). The CAP is initiated via activation of the catecholaminergic splenic nerve and release of norepinephrine (NE) and culminates with α7nAChR activation and inhibition of inflammation. Vagus nerve stimulation (VNS) also protects the kidney from injury (Abe et al. 2017; Inoue et al. 2016).

Our understanding, but still incomplete knowledge, of modulation of disease and tissue injury through the anti-inflammatory reflex pathway has become increasingly complex with research involving different animal models of inflammation. These models are invaluable as they begin to contribute to a unifying mechanism by which VNS (and other methods of stimulating the pathway) leads to an anti-inflammatory and tissue protective effect. In this review we describe disease models that contribute to our understanding of how VNS attenuates inflammation and organ injury: acute kidney injury, rheumatoid arthritis, and inflammatory gastrointestinal (GI) disease. We expand on the role of sympathetic efferent pathways in the inflammatory reflex that modulates these diseases. Lastly, we look at the synergy in these diseases by highlighting common neuronal pathways in the anti-inflammatory reflex (including new findings that challenge and expand the canonical view of the CAP) and by examining the interplay between chronic kidney disease (CKD), gut dysbiosis, and the CAP, which can lead to CKD progression.

### Inflammatory reflex and the cholinergic anti-inflammatory pathway (CAP)

**Fig. 1:**
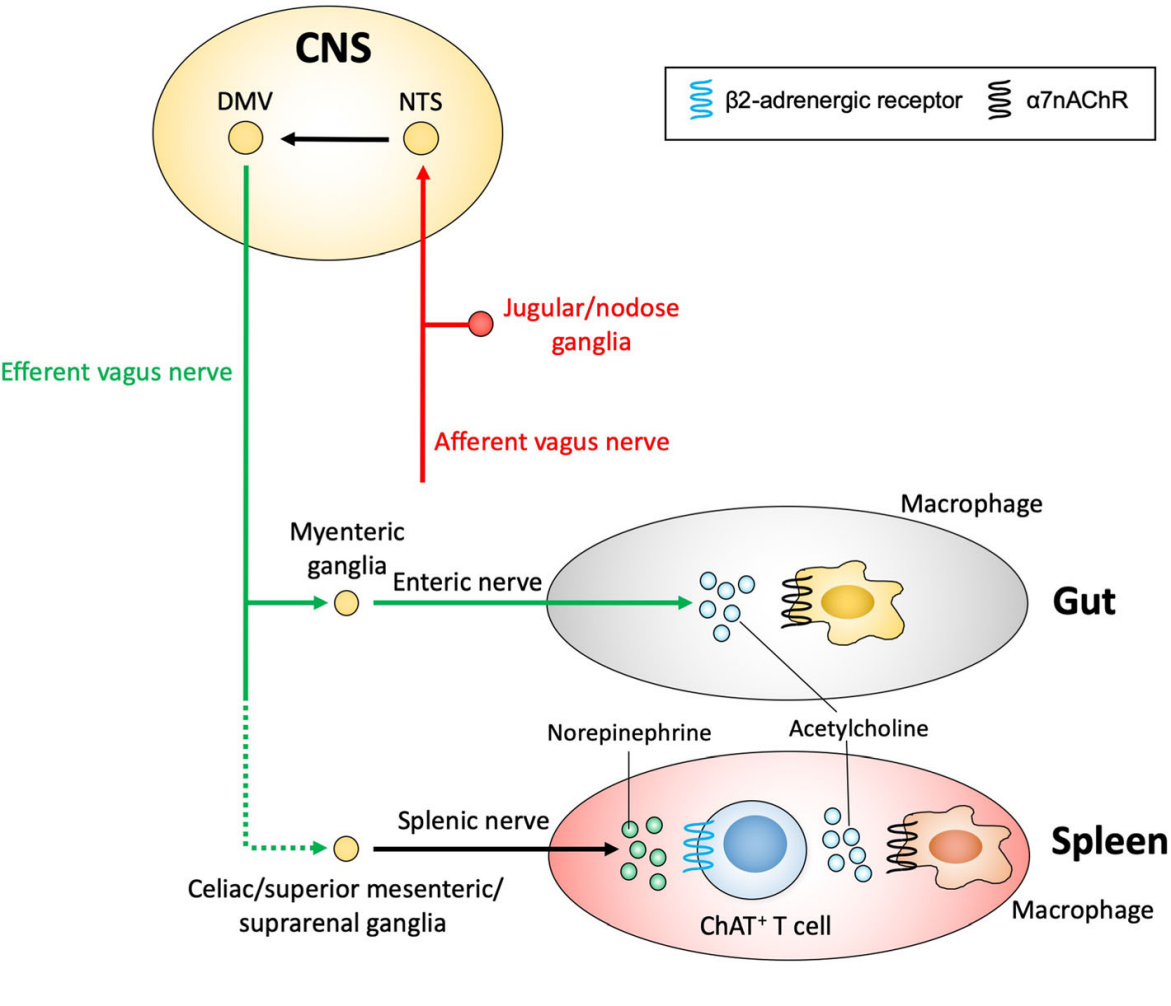
The inflammatory reflex. The inflammatory reflex is triggered when the afferent vagus nerve senses inflammatory products through the receptors. The nerve activity is relayed through the central nervous system (CNS) to the efferent vagus nerve. The original pathway involves the splenic nerve although a direct connection between the efferent vagus nerve and the splenic nerve is still controversial. Activated splenic nerves release norepinephrine from their terminals, which interacts with β2-adrenergic receptors expressed on the choline acetyltransferase (ChAT)-positive T cells in the spleen, causing acetylcholine (ACh) release from this specific T cell subpopulation. ACh binds to α7 nicotinic acetylcholine receptors (α7nAChRs) expressed on macrophages residing in close proximity to ChAT-positive T cells, resulting in suppression of proinflammatory cytokine production (e.g., TNFα) by macrophages and alleviated inflammation in many pathological settings (e.g., endotoxemia, acute kidney injury). Recent studies also suggested that a direct interaction between cholinergic enteric neurons and gut resident macrophages via ACh had an anti-inflammatory effect. DMV, dorsal motor nucleus of the vagus; NTS, nucleus tractus solitarius

The first clue for the existence of the inflammatory reflex was a seminal study by Linda Watkins (Watkins et al. 1995). She demonstrated that interleukin (IL)-1β-induced hyperthermia was blocked by subdiaphragmatic vagus transection, which indicated that the vagus nerve can sense inflammation at the periphery and send a signal to the central nervous system (CNS). Subsequently, Tracey found that a small amount of CNI-1493 (a potent anti-inflammatory agent) administered intracerebroventricularly significantly decreased lipopolysaccharide (LPS)-induced increases in levels of plasma tumor necrosis factor (TNF), which mainly originates from the spleen (Bernik et al. 2002). He further demonstrated that cutting the vagus nerve abolished the decrease in plasma TNF level by CNI-1493 administration and that electrical stimulation of the vagus nerve significantly decreased plasma TNF, suggesting that an anti-inflammatory signal can descend from the CNS through the vagus nerve to the spleen to alleviate peripheral inflammation. These findings formed the basis of the inflammatory reflex, where the afferent vagus nerve senses inflammation in the periphery, and the signal is transmitted to the efferent vagus nerve and the spleen to abrogate peripheral inflammation (Fig. 1) (Tracey 2007).

The inflammatory reflex is triggered when the afferent vagus nerve senses inflammatory products, such as cytokines, damage-associated molecular patterns, and pathogen-associated molecular patterns, through cytokine receptors and pattern-recognition receptors (Hosoi et al. 2005). The nerve activity is relayed through the CNS to the efferent vagus nerve and then to the splenic nerve (Rosas-Ballina et al. 2008). A direct connection between the efferent vagus nerve and the splenic nerve is still controversial (Martelli et al. 2014), although the celiac/superior mesenteric/suprarenal ganglia may connect these two nerves (Bellinger et al. 1989; Berthoud and Powley 1993; Berthoud and Powley 1996; Li et al. 2010; Nance and Burns 1989). Activated splenic nerves release NE from their terminals, which interacts with β2-adrenergic receptors expressed on the choline acetyltransferase (ChAT)-positive T cells in the spleen, causing acetylcholine (ACh) release from this specific T cell subpopulation (Rosas-Ballina et al. 2011). ACh binds to α7nAChRs expressed on macrophages residing in close proximity to ChAT-positive T cells, resulting in suppression of proinflammatory cytokine production (e.g., TNFα) by macrophages and alleviated inflammation (Rosas-Ballina et al. 2008; Wang et al. 2003). Intracellular mechanisms downstream of α7nAChRs include suppression of the NF-κB pathway (Guarini et al. 2003; Altavilla et al. 2006; Sun et al. 2013; Yoshikawa et al. 2006), activation of the Janus kinase 2/signal transducer and activator of transcription 3 (JAK2/STAT3) pathway (de Jonge et al. 2005; Lu et al. 2017), and inhibition of the NLR family, pyrin domain-containing 3 (NLRP3) inflammasome by preventing mitochondrial DNA release (Lu et al. 2014). The efferent arm of the inflammatory reflex was termed the cholinergic anti-inflammatory pathway (CAP) (Tracey 2007).

Although many studies have described the protective effects of efferent VNS via activation of the CAP, others have challenged and expanded the original view of the CAP both in terms of the efferent pathway and by beginning to define different pathways by which afferent VNS can attenuate inflammation (Martelli et al. 2014; Inoue et al. 2019). Moreover, the interaction between these neural circuits and the immune system seems to be very complex. Carnevale et al. demonstrated that T cells egress from the spleen and infiltrate the kidney and aorta to cause angiotensin II-induced hypertension and that the vagus nerve and splenic nerve are involved in these steps based on the experiments with vagotomy, celiac ganglionectomy, or selective splenic denervation (Carnevale et al. 2016; Carnevale et al. 2014). Increased splenic sympathetic nerve activity is also important in myeloid progenitor proliferation and differentiation in the spleen and the subsequent formation of atherosclerotic plaque in diabetes (Vasamsetti et al. 2018). Thus, the effect of the inflammatory reflex on the spleen and immune cells is complex and highly context-dependent.

### Acute kidney injury, neuromodulation of injury, and the CAP

Acute kidney injury (AKI) is a serious world-wide clinical concern since the incidence rate is high and AKI is associated with high mortality and morbidity. Moreover, AKI episodes can lead to CKD and end stage renal disease (Coca et al. 2012; Lassnigg et al. 2004; Tanaka et al. 2014). It is a general consensus that inflammation by immune cells such as neutrophils and mononuclear phagocytic cells plays a critical role in the pathophysiology of AKI (for reviews see (Li and Okusa 2010; Kinsey and Okusa 2012; Bonventre and Yang 2011; Rabb et al. 2016; Jang and Rabb 2015)), however pharmacological approaches that alleviate inflammation in AKI have been unsuccessful in clinical trials and no approved pharmacological agents are available to treat human AKI. Thus neuroimmunomodulation by non-pharmacological approaches is attracting considerable attention as an innovative alternative therapeutic strategy for AKI (Gigliotti et al. 2013; Abe et al. 2017; Inoue et al. 2016; Inoue et al. 2019; Tanaka et al. 2017; Gigliotti et al. 2015).

The kidney is densely innervated by sympathetic nerves, which reach all portions of the renal vessels and some tubules (Barajas et al. 1992). The sympathetic nervous system is well known to control factors important for blood pressure regulation such as heart rate and vascular tone, central nervous system regulation of cardiovascular function, sodium handling, and renin secretion (Dampney 2016; DiBona and Kopp 1997; Guyenet 2006; Joyner et al. 2010; Coffman 2014) An increase in efferent renal sympathetic nerve activity decreases renal blood flow (via α_1A_-adrenergic receptors on renal arterial vessels), decreases urinary sodium excretion (via α_1B_-adrenergic receptors on tubular epithelial cells), and increases renin secretion (via β1-adrenergic receptors on juxtaglomerular granular cells). Increased sympathetic activity to the kidney in hypertension forms the basis for the experimental and clinical use of renal denervation for blood-pressure lowering, but the contribution from renal sensory afferents in the renorenal reflex is not well understood (DiBona and Esler 2010). On the other hand, the innervation by sensory nerves in the kidney is limited predominantly to the pelvic region (Marfurt and Echtenkamp 1991). Based on these important functions mediated by renal sympathetic and sensory nerves, renal denervation for the treatment of resistant human hypertension is undergoing investigation and its efficacy remains controversial (Bhatt et al. 2014; Esler et al. 2012; Symplicity 2011).

Renal denervation has been tested in various kidney disease models including AKI. Renal denervation was protective in a rat model of anti-Thy-1.1 nephritis (Veelken et al. 2008) and a mouse model of lupus nephritis (Mathis et al. 2013). Renal denervation performed at 5 min (Fujii et al. 2003) or 2 h (Ogawa et al. 2002) before IRI also ameliorated AKI in rats. In these studies both renal sympathetic and sensory nerves were ablated, whereas Kim et al. investigated the roles of these nerves separately in a mouse model of unilateral ureteral obstruction (Kim and Padanilam 2013) and unilateral renal IRI (Kim and Padanilam 2015). Renal denervation significantly reduced the infiltration of neutrophils and macrophages into injured kidneys and renal fibrosis in these models. Notably, continuous infusion of norepinephrine or calcitonin gene-related peptide (CGRP) into the cortical region of the denervated kidney abrogated the protective effect of renal denervation in a dose-dependent manner, whereas infusion of neuropeptide Y or substance P did not have any effect. Furthermore α_2_-adrenergic and CGRP receptors, which are expressed in tubular epithelial cells, were necessary for the induction of renal inflammation and fibrosis. These findings indicate that both adrenergic signaling (sympathetic nerves) and CGRP signaling (sensory nerves) in the kidney play important roles in kidney inflammation and subsequent fibrosis.

Activation of the CAP is protective against AKI. Electrical stimulation of the left cervical vagus nerve 24 h before renal IRI significantly attenuated AKI in mice (Inoue et al. 2016). The protective effect was nullified in *α7nAChR*^*−/−*^ mice or splenectomized mice, which suggests that the protection by VNS is mediated by CAP activation (Fig. 1). Interestingly, the stimulation of either the peripheral or central end of the cut vagus nerve was sufficient to protect the kidneys. The stimulation of the central end (afferent VNS) was still protective when nerve conduction of the right vagus nerve was blocked by bupivacaine. These findings indicate that mechanisms other than vagovagal reflex exist in the protective effect of afferent VNS in AKI. VNS was also shown to be beneficial in renal transplantation (Hoeger et al. 2010; Hoeger et al. 2014). VNS in brain-dead donor rats attenuated inflammation in the donors and decreased immune cell infiltration to the recipient kidneys, improving long-term renal function and survival of the recipients.

Pulsed US may be a promising non-invasive approach to activate the CAP and protect the kidneys (Gigliotti et al. 2013; Gigliotti et al. 2015). US insonation with a clinical machine 24 h before IRI ameliorated AKI in mice. In splenectomized mice, mice with splenic sympathectomy, *α7nAChR*^*−/−*^ mice, mice treated with an antagonist of α7nAChR, and *Rag1*^*−/−*^ mice, US was not effective. Moreover, bone marrow chimera studies demonstrated that α7nAChRs expressed in hematopoietic cells are essential for the protection by US. Wasilczuk et al. (Wasilczuk et al. 2018) recently showed that focused ultrasound application to the left cervical vagus nerve in rats significantly reduced plasma TNFα levels after LPS administration, whereas US insonation targeted to the spleen also conferred an anti-inflammatory effect (Cotero et al. 2019) (Zachs et al. 2019), indicating that the vagus/splenic nerve and splenocytes can be the direct target of US. Regarding the mechanism by which US can affect the nerve, several possibilities have been proposed including a mechanical or thermal effect on mechanically-activated ion channels or voltage-gated ion channels expressed in the neurons and a cavitational effect leading to ionic flux (Downs et al. 2018; Kim et al. 2012; Tyler et al. 2008) (Wright et al. 2017). One intriguing possibility is that PIEZO1 and PIEZO2, mechanosensitive ion channels, could be involved, since these channels are expressed in the afferent vagus nerve (Zeng et al. 2018; Nonomura et al. 2017).

Abe et al. (Abe et al. 2017) explored the roles of restraint stress and C1 neurons in the context of CAP activation and AKI. C1 neurons located in the medulla oblongata innervate the dorsal motor nucleus of the vagus (DMV), sympathetic efferent pathways, the paraventricular nucleus of the hypothalamus, and other areas of the brain, and play a pivotal role in mediating autonomic responses to various stressors such as hypoxia and hypotension (Guyenet et al. 2013). Optogenetic stimulation of C1 neurons was protective against renal IRI in mice, and the effect was dependent on the spleen, α7nAChRs, and β2-adrenergic receptors. They also investigated the downstream pathway of C1 stimulation by performing ganglionic blockade, subdiaphragmatic vagotomy, and corticosterone receptor blockade. Only ganglionic blockade significantly attenuated the protective effect of C1 stimulation. Interestingly, physical restraint for 10 min was also protective against renal IRI, and the protection was blocked when C1 neurons were selectively ablated or inhibited. Taken together, these findings suggest that restraint stress can activate C1 neurons and the CAP, not through vagal efferents or the hypothalamic-pituitary-adrenal axis but through sympathetic efferents, leading to kidney protection. The terminal fields of these sympathetic efferents that protect the kidney have not yet been identified, nor has the specific mechanism of protection.

Another non-canonical CAP involves α7nAChR-positive peritoneal macrophages (Inoue et al. 2019). VNS or pulsed US changed the phenotype of α7nAChR-positive peritoneal macrophages, and adoptive transfer of these peritoneal macrophages protected recipient mice against renal IRI. The protective effect was not observed in *α7nAChR*^*−/−*^ mice, splenectomized mice, or *Rag1*^*−/−*^ mice lacking T and B cells. These results may suggest an interaction between α7nAChR-positive peritoneal macrophages and other immune cells including β2-adrenergic receptor-positive CD4+ T cells in the spleen. Pulsed US can target peritoneal macrophages directly or the vagus nerve existing in the peritoneum, considering that unfocused pulsed US was applied from the back using a big transducer in this study and that the distance between the vagus nerve and peritoneal cavity is small (Matteoli and Boeckxstaens 2013; Tanaka et al. 2002). The downstream signaling of α7nAChR in peritoneal macrophages was also investigated in this study. RNA sequencing of nicotine/LPS-treated peritoneal macrophages isolated from wild type and *α7nAChR*^*−/−*^ mice identified hairy and enhancer of split-1 (Hes1) as a key molecule to activate the CAP. Hes1, which is a transcriptional repressor, was reported to suppress production of inflammatory cytokines and chemokines in macrophages (Hu et al. 2008; Shang et al. 2016). Hes1 expression was induced in peritoneal macrophages by VNS or pulsed US, and adoptive transfer of Hes1-overexpressing peritoneal macrophages reduced renal IRI. These findings demonstrate that α7nAChR-positive peritoneal macrophages are involved in a non-canonical CAP and that Hes1 plays an important role in activating the CAP to protect the kidneys from injury.

### Rheumatoid arthritis

Rheumatoid arthritis (RA) is a chronic inflammatory disease that primarily affects joints (Smolen et al. 2018). Symptoms include painful swollen joints and ultimately bone destruction without effective treatment (Smolen et al. 2016). Patients with RA have a mortality rate 1.5- to1.6-fold higher than the general population (Sokka et al. 2008). Pharmacological treatments include TNF blocking agents and anti-IL-6 (Smolen et al. 2016). However, patients rarely reach remission with the current treatments (Tak and Kalden 2011).

Given the lack of effective therapeutic options, recent research has focused on other avenues of inflammation suppression such as neuromodulation. A study by Koopman et al. demonstrated the possibility of changes in the autonomic nervous system contributing to the onset of RA (Koopman et al. 2016). Elevation of resting heart rate in subjects at risk of RA indicated changes in autonomic balance before the development of RA. This, along with the recorded decrease in heart rate variability in RA subjects, suggests greater sympathetic control in the development of immune-mediated disease (Evrengul et al. 2004). The control that the vagus nerve has within the autonomic nervous system serves as a potential target for decreasing inflammation. Recent studies have demonstrated how the vagus nerve and the CAP suppress the progression of RA.

To use the vagus nerve’s protective effect on RA, electrical stimulation activates the CAP and induces a reduction in inflammatory cytokines. In a model of collagen-induced arthritis, rats subjected to VNS had a 52% reduction in swelling of the ankle (Levine et al. 2014). Additionally, levels of the inflammatory cytokines IL-1β, IL-2, IL-6, and TNF were reduced in VNS subjects.

To solidify these findings, Koopman et al. (Koopman et al. 2016) electrically stimulated the cervical vagus nerve and found marked attenuation of RA symptoms and inflammation in human subjects. The production of inflammatory cytokines was inhibited in subjects with VNS. In addition to the suppression of cytokine levels, disease severity was significantly attenuated. These findings indicate a pathway by which VNS can suppress the symptoms and progression of RA.

**Fig. 2:**
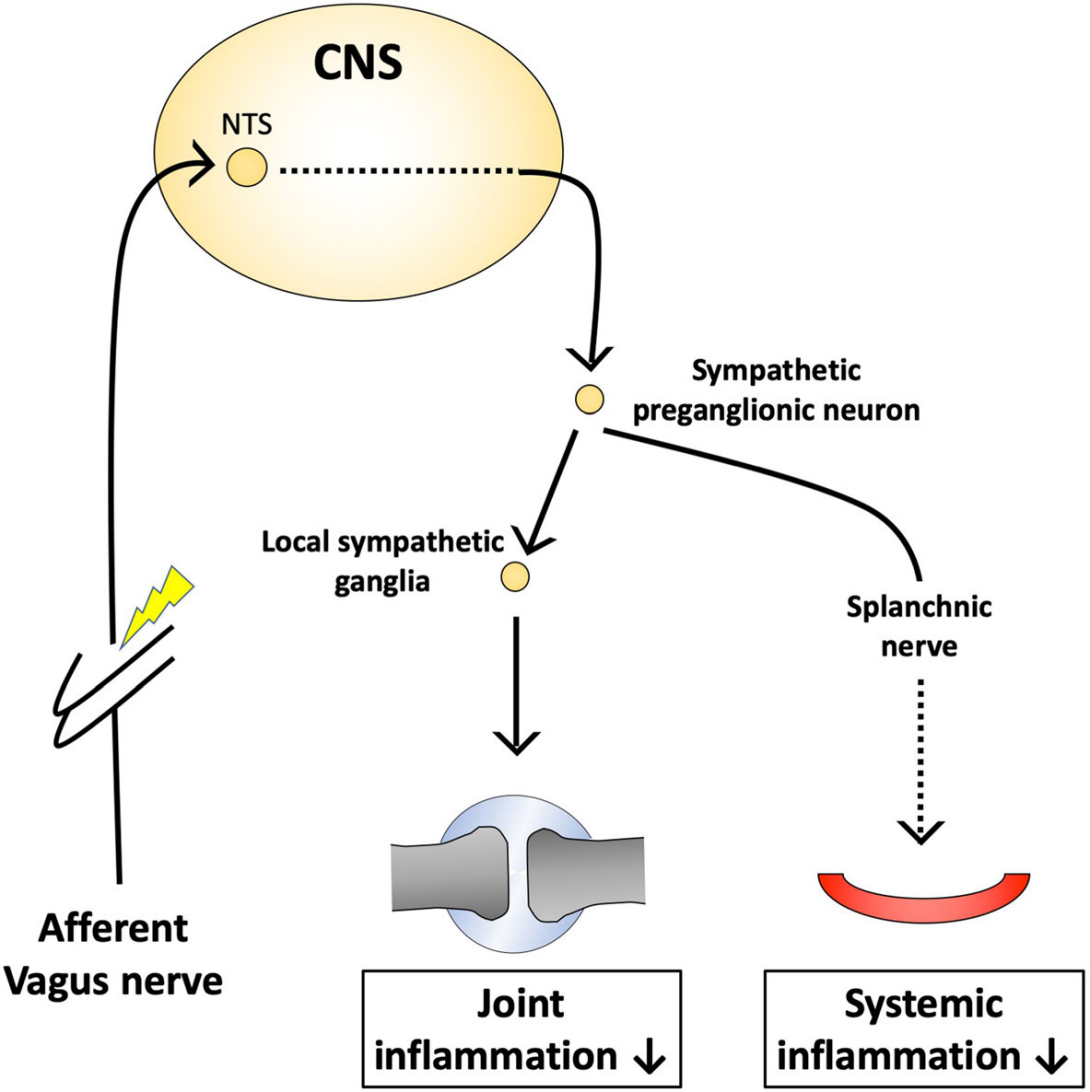
Another anti-inflammatory pathway elicited by afferent vagus nerve stimulation. Afferent vagus nerve stimulation can elicit an anti-inflammatory pathway involving sympathetic efferents through the central nervous system (CNS). In a model of joint inflammation, the local release of norepinephrine from sympathetic nerve terminals within joints alleviates inflammation. On the other hand, the splanchnic sympathetic nerve seems to be important to suppress systemic inflammation after lipopolysaccharide administration. Direct target(s) of the splanchnic nerve is not clear. NTS, nucleus tractus solitarius

Electrical stimulation of the cervical vagus activates both efferent and afferent vagus nerve fibers. Using a different method of neuromodulation, Bassi et al. (Bassi et al. 2017) stimulated the afferent fibers of the vagus nerve to attenuate RA. In subjects treated with afferent VNS, joint inflammation was suppressed. However, it was determined that this method of controlling inflammation is dependent on the local sympathetic nerves, which is a novel finding (Fig. 2). Although most studies focus on stimulation of the efferent vagus nerve pathway and its systemic effect (Bassi et al. 2017), Bassi et al. outlined mechanisms by which the treatment can locally regulate inflammation through sympathetic networks. They also showed that afferent VNS alleviated arthritic joint inflammation (Bassi et al. 2017). Notably, the protective effect persisted after splenectomy or subdiaphragmatic vagotomy, which suggests that the mechanism is different from the CAP. They performed additional experiments and concluded that afferent VNS activates sympathetic efferents through the CNS, which causes the local release of norepinephrine from sympathetic nerve terminals within joints, resulting in local regulation of an innate immune response (Bassi et al. 2017). Another group also demonstrated that activating abdominal vagal afferent fibers suppressed systemic inflammation after LPS administration and that the efferent arm of this pathway is in the splanchnic sympathetic nerves (Komegae et al. 2018).

To summarize, the attenuation of RA inflammation through neuromodulation, specifically VNS, has been found in many recent studies. However, as a topic that requires further studying Bassi et al. (Bassi et al. 2017) outlined an afferent pathway that locally suppresses symptoms of RA through the activation of sympathetic efferents (Fig. 2). Future research should focus on specific neural circuits that control inflammation in models of arthritis.

### Neuromodulation of gastrointestinal (GI) disorders using bioelectronics

Crohn’s disease and ulcerative colitis, which are grouped together in the category inflammatory bowel disease (IBD), are painful and debilitating chronic inflammatory disorders of the gut that affect ~ 1.6 million people in the US and are characterized by dysregulated responses to the gastrointestinal microbiome. Symptoms include diarrhea, abdominal pain, fatigue, weight loss, and others. Anti-inflammatory and immunosuppressive treatments typically used for IBD have numerous side effects, and with chronic treatment they lose their effectiveness over time in some patients.

The anatomy and physiology of the GI tract, as well as the inflammatory profile of many GI disorders, makes VNS (or in some cases other neuromodulatory modalities) an attractive candidate as an alternative to existing therapies. As in other diseases and disorders for which VNS is currently approved as a treatment or is in clinical trials, the strategy is to employ neural circuitry to dampen inflammation in target organs by stimulating anti-inflammatory pathways. The vagus (both afferent and efferent vagus nerve fibers), thoracolumbar and sacral nerves innervate the gut (stomach and intestines). Experimental and clinical findings on the role of these neuronal pathways of the autonomic nervous system in regulating gastrointestinal function in normal health and disease provide an anatomical and physiological rationale for neuromodulation of GI disorders (Payne et al. 2019). For example, blunted vagus nerve tone associated with a pro-inflammatory profile has been observed in patients with IBD (Pellissier et al. 2014) and suggests that a neuronal imbalance could be causative at least in part for disease manifestation. Reduced vagus nerve activity would impact the CAP and could lead to elevated proinflammatory factors (e.g, TNFα, which is high in IBD patients) and conversely VNS of a suboptimally stimulated system could restore downstream pathways in the CAP, such as reducing TNFα release from macrophages systemically or from gut resident macrophages (Fig. 1).

In preclinical studies VNS reduced inflammation in trinitrobenzenesulfonic acid (TNBS)-induced colitis, an experimental model of Crohn’s Disease in rats (Meregnani et al. 2011) (Sun et al. 2013) (Jin et al. 2017). VNS also improved survival and reduced inflammation in oxazolone-induced colitis in mice, a model that is similar to severe ulcerative colitis (Meroni et al. 2018). Small pilot clinical studies have examined effectiveness of VNS in patients with Crohn’s disease, including those patients refractory to treatment. In the first study nine Crohn’s patients were implanted with vagus nerve stimulators and stimulated chronically. Remission occurred in 5 of 9 patients (based on clinical score, biological measures, endoscopic findings) and vagal tone was restored (Bonaz et al. 2016). While this study is promising, some patients did not respond, inclusion was complicated to some degree by variability of prior drug treatment (and whether patients were undergoing standard care at the time of the trial), and larger carefully controlled randomized trials are needed.

Some of the clinical trials of bioelectronic neuromodulation for other gastrointestinal and gastrointestinal-related disorders, which may not in every case be targeting inflammatory parameters, have been reviewed recently (Payne et al. 2019). These include trials on irritable bowel syndrome (ClinicalTrials.gov Identifier: NCT02388269; NCT02420158), postoperative ileus (disrupted GI motility after abdominal surgery), obesity, gastroparesis, and colorectal dysfunction (incontinence or constipation), and manipulations include blocking or stimulating the vagus, sacral nerve stimulation, and gastric electrical stimulation. A search of the keywords gastrointestinal and neuromodulation in ClinicalTrials.gov returns 28 studies.

Treatment of GI diseases and disorders, like kidney injury or disease, with neuromodulatory approaches, and VNS in particular, faces a host of existing challenges. From the standpoint of patient acceptance and as yet undetermined relative treatment efficacy, invasive vs. non-invasive (transcutaneous stimulation of vagal auricular branch or cervical vagus) approaches need to be carefully evaluated; patients may be inclined to either resistance to or acceptance of surgical implantation. In the case of VNS for depression and epilepsy, stimulation parameters (e.g., frequency and duration) have seen FDA approval, but a careful analysis of efficacy vs. side effects is needed for each new use. One approach to avoiding surgical implantation has been to explore the effectiveness of transcutaneous stimulation of either the cervical vagus or transauricular branch of the vagus but rigorous comparisons must be made to establish efficacy.

Finally, the goal of treatment of IBD is not only to reduce symptoms but also to promote healing of the mucosal lining of gut and reestablish homeostasis. Careful studies will be needed to determine if chronic anti-inflammatory strategies, or other physiological effects, mediated by neuromodulation play a role in the repair process and reversal of dysbiosis.

### Organ crosstalk: gut microbiota and the CAP in progression of CKD

In the preceding sections, we reviewed the inflammatory reflex pathway and its modulation with VNS to reduce inflammation and preserve or improve function in acute kidney injury, rheumatoid arthritis, and inflammatory gastrointestinal disease. While these diseases share the commonality of inflammation, they may otherwise seem unrelated. However, interaction between distant tissues can be important both physiologically and pathologically. Normal homeostatic function is maintained by a complex biological system of crosstalk between organs, but this organ crosstalk can contribute to the detrimental effects of disease in one organ on the functional state of distant organs. Organ crosstalk has been described in a variety of diseases, including acute kidney injury (Lee et al. 2018) and cardiorenal syndrome (Virzi et al. 2014). The CAP, by linking nervous and immune systems to modulate end organ inflammation, is an example of one avenue of communication between various organ systems. Future studies will be needed to better define and maximize the efficacy of bioelectronic medicine as it relates to the vagus vis-à-vis organ crosstalk, as it is possible that manipulations such as VNS could benefit multiple organs. In this section, we aim to provide a perspective on neuromodulation of the gastrointestinal system at the level of the gut microbiome and how organ crosstalk between the gut and kidney, including effects of CKD on the gut microbiome, could be potentially modulated by VNS to alter the progression of CKD.

While gut microbiota, the diverse and numerous microbes that reside in the gastrointestinal tract, are known to be critical to normal health, it is increasingly recognized that gut dysbiosis, a condition of imbalance in the normal microbiome, plays a role in a variety of diseases, especially in those with an inflammatory component including obesity (Kang and Cai 2017; Turnbaugh et al. 2006), diabetes mellitus (Weiss and Hennet 2017; Forslund et al. 2015), asthma (Kang et al. 2017; Saltzman et al. 2018), heart failure (Lau and Vaziri 2017), cancer (Raskov et al. 2017; Dejea et al. 2018), IBD (Moustafa et al. 2018), non-alcoholic fatty liver disease (Saltzman et al. 2018; Henao-Mejia et al. 2012), chronic kidney disease (Lau and Vaziri 2017; Noel et al. 2014; Jazani et al. 2019; Nigam and Bush 2019; Yang et al. 2018), major depressive disorders (Zheng et al. 2016) and cardiovascular disease (Carding et al. 2015).

The composition and function of commensal microorganisms can be altered by foreign pathogens and other environmental factors (such as diet, toxins and stress). Resident microorganisms in the gut are essential for normal physiological processes such as development of the immune system and metabolic regulation. Still, the host must be protected from systemic invasion by both commensal and pathogenic microorganisms, and this occurs by a process that involves inherent physical, chemical and biological barriers in the gut, including specialized intestinal epithelial and immune cells and carefully orchestrated local and systemic interactions with the immune system. Intestinal permeability is essential for fluid transport and nutrient uptake but permeability and barrier function can be disrupted by host disease and can contribute to a variety of inflammatory disorders. The bidirectional interplay between local intestinal flora, the gut epithelial barrier, and immunomodulatory processes can impact other organs and disease progression and vice versa. We highlight here the interactions between the microbiome, the immune system, and the kidney in CKD and at this point in time, hypothetical but intriguing possibilities for targeting mechanisms in the immune reflex pathway to modulate these interactions.

CKD contributes to gut dysbiosis, including a profound change in the composition of the gut microbiome in patients with CKD or end stage renal disease and in models of CKD in rats (Vaziri et al. 2013) and altered intestinal permeability (Knauf et al. 2019). Each of these can have deleterious outcomes on CKD progression by exacerbating chronic inflammation. Metabolic and immune pathways respond to the gut microbiome, communicate with each other, and participate in kidney-gut crosstalk. In the face of dysbiosis gut-microbiota-kidney crosstalk both responds to and contributes to CKD.

The relationship between the gut and kidney is influenced by two pathways: 1) metabolism-dependent and 2) immune-dependent pathways. Diet is important in the gut microbiome (Gong et al. 2019); diets that are high in fat and protein and low in fiber can lead to the generation of “uremic toxins” by the gut microbiota (Sirich et al. 2014). Two solutes, indoxyl sulfate and p-cresol sulfate, thought to be uremic toxins, were reduced in hemodialysis patients by ~ 30% when patients were placed on a high fiber diet. These toxins are thought to be derived from the colon as patients on hemodialysis who had intact colons had higher levels of uremic toxins than those who did not have colons (Aronov et al. 2011). The gut microbiota also influences immunity. Bacterial products can activate toll-like receptors on dendritic cells that induce T regulatory cells to produce Il-10. Short chain fatty acids are ligands for G protein-coupled receptor 43 (GPCR43) that is expressed on intestinal epithelial cells, regulate T regulatory cells and control barrier function by regulating tight junctions (Knauf et al. 2019).

The vagus nerve plays a central role in the communication between the brain and the gut microbiota. Vagus nerve afferents are distributed to all layers of the digestive wall but do not penetrate to the lumen (Wang and Powley 2007). Thus communication between gut microbiota to the vagus afferents is indirect through diffusion of bacterial products or metabolites (Wang and Powley 2007). Activation of the vagal afferents by gut microbiota leads to increased neuronal activity in the nucleus tractus solitarius (NTS) followed by widespread activation of the autonomic network (Good et al. 2018; Bonaz et al. 2018). VNS leads to effects on gut inflammation/immunity as well as epithelial permeability, but there is no evidence yet that VNS alters gut microbiota (Bonaz et al. 2018).

## Conclusions

The inflammatory reflex pathway consists of afferent and efferent fibers of the vagus nerve as well as sympathetic efferents that serve as a communication network whereby the peripheral afferents can sense changes in the local milieu sending information to the brain and reflex signals to curtail inflammation. The inflammatory reflex pathway provides an opportunity to develop novel pharmacological and nonpharmacological approaches. The advantage of the latter is the ability to avoid lack of specificity and off target effect of drugs. We have highlighted the potential use of bioelectronic approaches and the biological basis for activation of the inflammatory reflex pathway in curtailing inflammation in various inflammatory conditions. Further refinements in bioelectronics continue to be developed. For example, minimally invasive, targeted use of an ultrasound-guided needle electrode can be used to stimulate the vagus nerve (Huffman et al. 2019). Early clinical trials are encouraging, however much work needs to be done to define specific circuits that will improve precision.

## Data Availability

Not applicable.

## Abbreviations

ACh: Acetylcholine
AKI: Acute kidney injury
CAP: Cholinergic anti-inflammatory pathway
CGRP: Calcitonin gene-related peptide
ChAT: Choline acetyltransferase
CKD: Chronic kidney disease
DMV: Dorsal motor nucleus of the vagus
GI: Gastrointestinal
IBD: Inflammatory bowel disease
IL: Interleukin
LPS: Lipopolysaccharide
RA: Rheumatoid arthritis
TNF: Tumor necrosis factor
US: Ultrasound
VNS: Vagus nerve stimulation
α7nAChR: Alpha-7 nicotinic acetylcholine receptor
